# The Epidemiology, Virology and Clinical Findings of Dengue Virus Infections in a Cohort of Indonesian Adults in Western Java

**DOI:** 10.1371/journal.pntd.0004390

**Published:** 2016-02-12

**Authors:** Herman Kosasih, Bachti Alisjahbana, Nurhayati, Quirijn de Mast, Irani F. Rudiman, Susana Widjaja, Ungke Antonjaya, Harli Novriani, Nugroho H. Susanto, Hadi Jusuf, Andre van der Ven, Charmagne G. Beckett, Patrick J. Blair, Timothy H. Burgess, Maya Williams, Kevin R. Porter

**Affiliations:** 1 Viral Diseases Program, U.S. Naval Medical Research Unit No. 2, Jakarta, Indonesia; 2 Health Research Unit, Faculty of Medicine, Universitas Padjadjaran, Bandung, Indonesia; 3 Department of General Internal Medicine, Radboud University Nijmegen Medical Centre, Nijmegen, The Netherlands; 4 Department of Internal Medicine, Hasan Sadikin Hospital, Bandung, Indonesia; 5 National Institute of Health Research and Development, Ministry of Health, Jakarta, Indonesia; University of California, Berkeley, UNITED STATES

## Abstract

**Background:**

Dengue has emerged as one of the most important infectious diseases in the last five decades. Evidence indicates the expansion of dengue virus endemic areas and consequently the exponential increase of dengue virus infections across the subtropics. The clinical manifestations of dengue virus infection include sudden fever, rash, headache, myalgia and in more serious cases, spontaneous bleeding. These manifestations occur in children as well as in adults. Defining the epidemiology of dengue in a given area is critical to understanding the disease and devising effective public health strategies.

**Methodology/Principal Findings:**

Here, we report the results from a prospective cohort study of 4380 adults in West Java, Indonesia, from 2000–2004 and 2006–2009. A total of 2167 febrile episodes were documented and dengue virus infections were confirmed by RT-PCR or serology in 268 cases (12.4%). The proportion ranged from 7.6 to 41.8% each year. The overall incidence rate of symptomatic dengue virus infections was 17.3 cases/1,000 person years and between September 2006 and April 2008 asymptomatic infections were 2.6 times more frequent than symptomatic infections. According to the 1997 WHO classification guidelines, there were 210 dengue fever cases, 53 dengue hemorrhagic fever cases (including one dengue shock syndrome case) and five unclassified cases. Evidence for sequential dengue virus infections was seen in six subjects. All four dengue virus serotypes circulated most years. Inapparent dengue virus infections were predominantly associated with DENV-4 infections.

**Conclusions/Significance:**

Dengue virus was responsible for a significant percentage of febrile illnesses in an adult population in West Java, Indonesia, and this percentage varied from year to year. The observed incidence rate during the study period was 43 times higher than the reported national or provincial rates during the same time period. A wide range of clinical severity was observed with most infections resulting in asymptomatic disease. The circulation of all four serotypes of dengue virus was observed in most years of the study.

## Author Summary

Dengue is the fastest spreading mosquito borne diseases in the world and is endemic in most tropical and sub-tropical countries with an estimated 96 million infections resulting in clinical disease annually. Population-based longitudinal prospective studies are essential for understanding dengue virus in the natural setting and developing prevention strategies to curtail the impact of disease. We present the results of a longitudinal cohort study of adults in West Java, Indonesia that was conducted between 2000–2004 and 2006–2009. We found that in adults, dengue virus was a significant cause of febrile illness. The entire spectrum of clinical severity was observed with most dengue virus infections manifesting as asymptomatic. The incidence of symptomatic dengue virus infections observed in this cohort was much higher than reported national or provincial rates. In addition, we observed all four dengue virus serotypes circulating during most years of the study.

## Introduction

Dengue is caused by infection with one of the four dengue viruses: dengue virus 1 (DENV-1), DENV-2, DENV-3 and DENV-4 [1]. Infection with any of these viruses may result in asymptomatic infection, dengue fever (DF), or the more severe forms, dengue hemorrhagic fever (DHF) and dengue shock syndrome (DSS). DHF and DSS were recognized in Southeast Asia soon after multiple serotypes began to circulate in the 1950s [2,3]. Since then the burden of dengue has increased rapidly with the number of annual cases worldwide rising from 908 in the 1950s to 925,896 in the 2000s[4]. The number of dengue-endemic countries has also expanded from nine to over 110[4,5]. Cases of DHF and DSS have also been increasingly recognized in other regions including South Asia, Latin America and the Pacific [6–9], with pediatric cases being more common. In recent years, DF and DHF/DSS have become more common in adults [10–12]. Because of the increased geographical circulation of the virus and the impact of the infection, dengue virus is widely recognized as the most important arboviral infection worldwide.

Many clinical and epidemiological studies on dengue have relied on outbreak investigations and hospital based studies [13–23]. These studies provide a wealth of data regarding clinical manifestations, laboratory parameters, pathology, and management of the disease. However, they also have some limitations. Hospital studies, for instance, mostly represent severe cases and do not cover the wide clinical spectrum of dengue infections in adults. Furthermore, hospital studies lack pre-illness and early illness sera that can be used to characterize an individual’s pre-infection dengue virus immune status or to measure laboratory predictors of disease severity. Therefore, there is a need for prospective population-based studies to complement the hospital-based investigations [24]. This form of study contributes to an overall picture of the spectrum of clinical disease in a given geographical area. To study the epidemiology of dengue virus in Bandung, West Java, Indonesia, we conducted a prospective study in a cohort of adults from August 2000 to June 2004 and from September 2006 to April 2009. The aims of the study were to determine the incidence of symptomatic and asymptomatic infections; determine the temporal distribution of dengue virus serotypes; characterize the clinical manifestations of dengue in adults and determine whether there is a correlation between severity of disease, pre-illness immune status and sequence of infections.

Preliminary results of the first two years were published previously [25]. Here, we report a comprehensive seven year account of the epidemiology, virology, immunology and clinical presentation of dengue virus infections within West Java cohorts.

## Materials and Methods

### Ethical considerations

The study protocol was reviewed and approved by the Institutional Review Boards at the U.S. Naval Medical Research Unit No. 2 and the National Institute of Health Research and Development, Ministry of Health, Indonesia (DoD 30855, KS.02.01.2.1.2181 and N2.2006.0001, KS.02.01.2.1.2776) in compliance with all U.S. Federal Regulations governing the protection of human subjects. Each volunteer provided informed written consent upon enrollment.

### Study design

**Fig 1:**
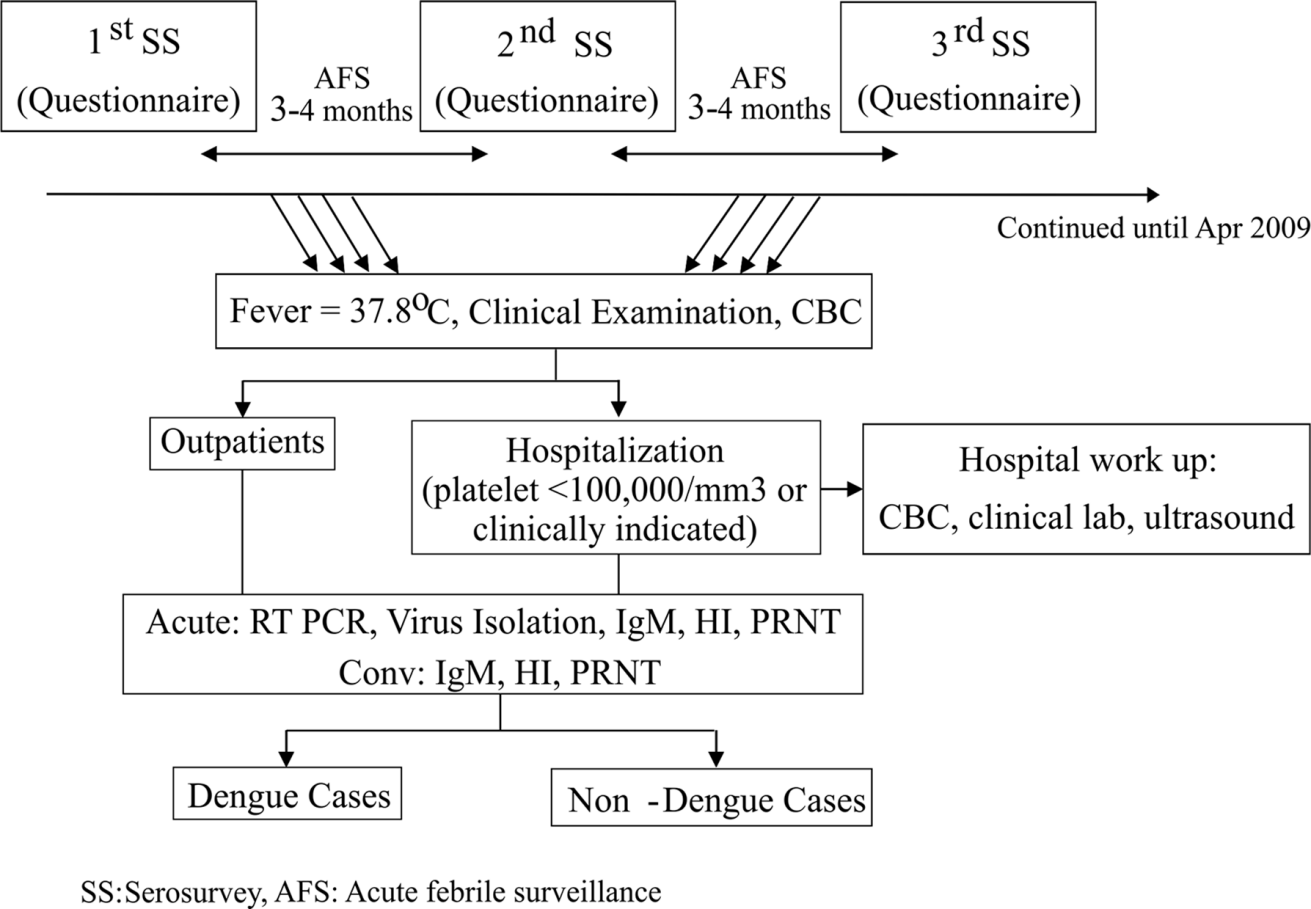
Overall study design. SS = serosurvey, AFS = acute febrile surveillance.

The study was conducted in two phases: from August 2000 to June 2004 and from September 2006 to April 2009. The first phase was carried out in factories A and B and the second phase in Factories A and C. Approximately 70% of the total factory workers participated in the study. A cohort of 2978 adult volunteers was prospectively followed during the first phase and 2726 during the second phase. Among these volunteers, 1324 participated in both phases. Details of the study design and procedures are illustrated in Fig 1 and are also described by Porter et al [25]. Briefly, blood was collected during enrollment and every three to four months thereafter. Factory employees who were ill were required to be seen by a factory clinician in order to be officially excused from work and still receive wages for the day. Employees who were absent, but did not report to the factory clinic were visited by a study team nurse. Volunteers who experienced fever were evaluated at the factory clinics for clinical assessment and blood was collected when indicated by study clinicians or nurses. A complete blood count (CBC) and dengue virus infection diagnostic tests as described below, were performed. Patients were advised to be hospitalized if their platelet count was less than 150,000/mm^3^ or at the discretion of the clinic attending physicians. In order to account for any illnesses that may have been missed, at each serosurvey volunteers were asked about any history of fever or any other illness since their last serosurvey (S1 Form). For volunteers meeting hospitalization criteria, clinical data was collected every day. For volunteers with confirmed dengue virus infection who did not meet hospitalization criteria, daily observation was conducted by study nurses either through home visits or phone calls.

### Dengue virus infection diagnostic assays

To diagnose dengue virus infection, virus isolation [25] and RT-PCR [26] were performed on blood specimens collected during the acute phase of illness. Dengue virus IgM, IgG antibody ELISA (FocusTechnology), and hemagglutination inhibition (HI) assays [27] were performed on acute and convalescent specimens. A plaque reduction neutralization test (PRNT) using BHK-21 cells was performed on pre-illness, acute and convalescent specimens from confirmed dengue virus infection patients. PRNT was also used on paired serosurvey specimens to confirm potential asymptomatic dengue virus infections identified using a screening strategy described below. The dilution that produced a 50% reduction in plaque count compared to a negative control sample was determined by probit analysis using SPSS.

### Asymptomatic dengue virus infections

To estimate the incidence of asymptomatic dengue virus infections, 25% of the total volunteer population from September 2006 to February 2008 (675 volunteers) was randomly selected using SPSS. Basic demographics (gender, age, factory of employment) between the randomly selected subset and the entire cohort during that time period were not statistically different. During this period, serum samples were collected every three to four months from each volunteer up to a total of six serum samples. In lieu of testing thousands of samples for which the HI test is too cumbersome, we developed a new method to identify asymptomatic dengue virus infections utilizing dengue virus IgG ELISA (Focus Technology) assays for screening followed by PRNT for confirmation. First, we established an IgG index ratio (IR) that could be used to identify potential asymptomatic infection cases from consecutive serosurvey samples. In order to do this, we tested 43 paired serosurvey sera, collected before and after confirmed symptomatic dengue virus infection episodes, along with 38 paired sera from confirmed non-dengue virus febrile episodes. A post-/pre-illness IgG IR was calculated in dengue virus and non-dengue virus febrile episodes and receiver operating characteristic (ROC) analysis was used to determine the IgG cut-off ratio to identify dengue virus infections. As samples identified through this screening process were to be further tested by PRNT, a conservative cut-off ratio was chosen in order to ensure that no cases were missed. We chose the lower IR between the lowest IR in the dengue virus infection group and the highest IR in non-dengue virus infection group as the cut-off value for screening. This resulted in an IR of 1.2. Of note, this strategy did not work if non-consecutive (more than 4 months between samples) serosurvey samples were used. In order to validate this approach, we ran IgG ELISAs on the 13 sample sets from the first two years of the study that were screened for asymptomatic infection using HI and confirmed by PRNT[25] and a random sampling of sample sets that were not identified as asymptomatic dengue virus infections based on HI screening (n = 47). For all confirmed asymptomatic dengue virus infections (≥ 4-fold increase in HI titer confirmed by PRNT), the IgG ratio between consecutive serosurvey samples was ≥1.2 (range 1.2 to 29.1) and in all cases tested that were not identified as asymptomatic dengue virus infections based on HI screening (<4-fold increase in HI titer), this ratio was less than 1.2 (range 0.9 to 1.1) (S1 Fig).

Upon validating the cut-off IgG IR, six serial serosurvey specimens from 675 volunteers were tested. Specimens with IgG IRs ≥ 1.2 between two consecutive serosurvey samples were further tested by PRNT. The serosurvey samples from each volunteer were tested simultaneously according to the manufacturer’s instructions, using the same lot of the dengue virus IgG ELISA kit.

### Sequence analysis

The envelope genes from eight DENV-1, one DENV-2, three DENV-3 and six DENV-4 isolates were sequenced (Genbank accession numbers KR604819-35) as previously described [28–30]. In brief, viral RNA was extracted from virus isolates using the Qiamp Viral RNA mini kit (Qiagen, Germany). Three overlapping fragments covering the Envelope-NS1 genes (approximately 2700 bases) were amplified by RT-PCR using serotype specific primer sets. Amplicons were purified and the BigDye cycle sequencing kit (Applies Biosystems, USA) was used for sequencing reactions. Sequencing reactions were run on a 3130 XL *Genetic Analyzer* (Applied Biosystems) and sequence outputs were assembled using Sequencher software (Genecodes, USA). Phylogenetic trees were generated using the Neighbor Joining method with bootstrapping in MEGA 4 [31].

### Definitions

The following definitions were used in this study:

**Dengue virus infection:** a recent dengue virus infection was confirmed when DEN virus was isolated, or the RNA was detected in an acute sample, and/or IgM seroconversion, and/or a four-fold or greater increase in HI antibody titers between acute and convalescent specimens was observed.

**Primary dengue virus infection:** a confirmed dengue virus infection in which dengue virus IgG antibodies were not detected in the acute sample and an HI titer of ≤1:80 in the convalescent specimen was observed. When indeterminate by IgG and HI, cases were also classified as primary infections when PRNT_50_ seroconversion to at least one serotype was detected between acute PRNT-negative and convalescent specimens.

**Secondary dengue virus infection:** a confirmed dengue virus infection in which dengue virus IgG antibodies or HI antibodies were detected in acute specimens or increased to ≥ 1:1280 in convalescent specimens. In instances indeterminate by IgG and HI, cases were classified as secondary infections when the presence of neutralization antibodies to any serotype in the acute specimens was detected.

**Tertiary dengue virus infection:** the second confirmed dengue virus infection during a volunteers participation in the study when the individual had clear evidence (by PRNT) of prior dengue virus infection upon enrollment in the study.

**Clinical category:** clinical data were analyzed using WHO 1997 criteria. Cases with evidence of plasma leakage (hematocrit increase ≥20%, or pleural effusion/acites by ultrasonogram), but no thrombocytopenia <100,000/mm^3^ were categorized as unclassified.

**Asymptomatic dengue virus infection:** An asymptomatic dengue virus infection was confirmed when there was no reported fever day or other illness and a four-fold or greater increase of PRNT_50_ titer in any serotype between two serosurvey specimens whose IgG IR ≥ 1.2 was observed. For primary asymptomatic cases, the infecting serotype was determined by the serotype with the highest PRNT_50_ titer in the second specimen.

**Naïve population**: a subset of study participants whose serosurvey specimens did not show antibodies to dengue as verified by IgG index <1.

**Pre-illness neutralizing antibody**: pre-illness neutralizing antibodies, as measured by PRNT, were present when the titer >1:10 and considered protective when the titer was >1:100 [32].

### Data analysis

Incidence of symptomatic and asymptomatic DEN virus infection was expressed as the number of infections occurring among the cohort per 1,000 person years of follow-up. Volunteers that dropped out from the study were accounted for in the denominator (total person-years) by including only the length of time they were available for follow-up. For comparison between two proportions, the chi-square test was used using STATA 9 software (Texas).

## Results

### Study population

The study was conducted in two phases: from August 2000 to June 2004 and from September 2006 to April 2009. During each phase of the study the goal was to maintain a cohort of approximately 3,000 volunteers. Over the course of both phases, a total of 4,380 volunteers from three factories were enrolled in the study. Factory A was included in both phases of the study and 1324 volunteers from Factory A participated in both phases of the study. Twenty percent of the volunteers participated in the study for more than six years, 19% participated for 3–4 years, 26.4% for 2–3 years and 16.9% for 1–2 years. The mean (SD) age of volunteers at enrollment was 35.6 (7.7) with a range between 18 and 66 years. A higher proportion of the study population was male (ratio 1.87: 1). Demographics of the study population aggregated by factory are shown in Table 1.

**Table 1:** Cohort demographics.

	Factory A		Factory B	Factory C	Total
Time period	AUG 00-JUN 04	AUG 06-APR 09	AUG 02-JUN 04	AUG 06-APR 09	
Males	1295 (64%)	911 (64%)	438 (46%)	1055 (81%)	2855 (65%)
Females	731	515	514	245	1525
Mean age at enrollment	36.9	36.2	31.9	36.2	35.6
Age range at enrollment	18–64	18–53	18–66	19–55	15–66

### Seroprevalence and asymptomatic dengue virus infections

The presence of asymptomatic infections was determined in 675 subjects randomly chosen from the September 2006 to February 2008 time period who did not experience an illness in between serosurveys. No evidence of a previous dengue virus infection was found in 15 (2.2%) of these subjects. Serological evidence of a previous dengue virus infection was found in 87.53% of subjects aged 18–27 years, in 96.6% aged 28–37 years, in 99.1% aged 38–47 years, and in all subjects above ≥48 years old. This trend was found to be statistically significant with prevalence higher in older age categories (Chi-squared test for trends, p<0.001).

Consecutive serosurvey samples were screened for asymptomatic infections using an IgG index ratio of ≥ 1.2 as a cut off. An IgG index ratio (IR) ≥ 1.2 between two consecutive serosurvey samples was found in 35 of 675 volunteers. After confirmatory PRNT testing, seven were excluded as no four-fold increase in any serotype was observed (of note: all of the excluded cases had low IgG IRs). Three of the 28 asymptomatic cases were primary infections, two were due to DENV-4 and one was due to DENV-1. We evaluated “pre-illness” PRNT titers for the 25 secondary infection asymptomatic cases. In four cases, the highest PRNT antibody titers to any serotype were below the level of the suggested protective neutralizing titer (≥1:100) [32]. In nine cases, protective neutralizing titers to one serotype were detected (five to DENV-3, two to DENV-1 and one each to DENV-2 and DENV-4). In eight cases, protective neutralizing titers were detected for two serotypes (six for DENV-1 and 3, one for DENV-1 and 2, and one for DENV-2 and 3) and protective neutralizing titers to three serotypes (DENV-1, 2 and 3) were identified in four cases. During the same time period, 43 symptomatic dengue virus infections occurred in the cohort resulting in an asymptomatic to symptomatic dengue virus infection ratio of 2.6:1 (95% CI:1.6–3.12). The average age of subjects with asymptomatic dengue virus infection was significantly higher than subjects with symptomatic dengue (40.14, 95% CI: 37.34–42.94 vs. 36.0, 95% CI: 35.06–36.94). The proportion of women with asymptomatic dengue virus infections was higher than the proportion with symptomatic dengue virus infections, but not significant (39.3% vs. 29.5%, p = 0.28).

### Symptomatic dengue virus infections

**Fig 2:**
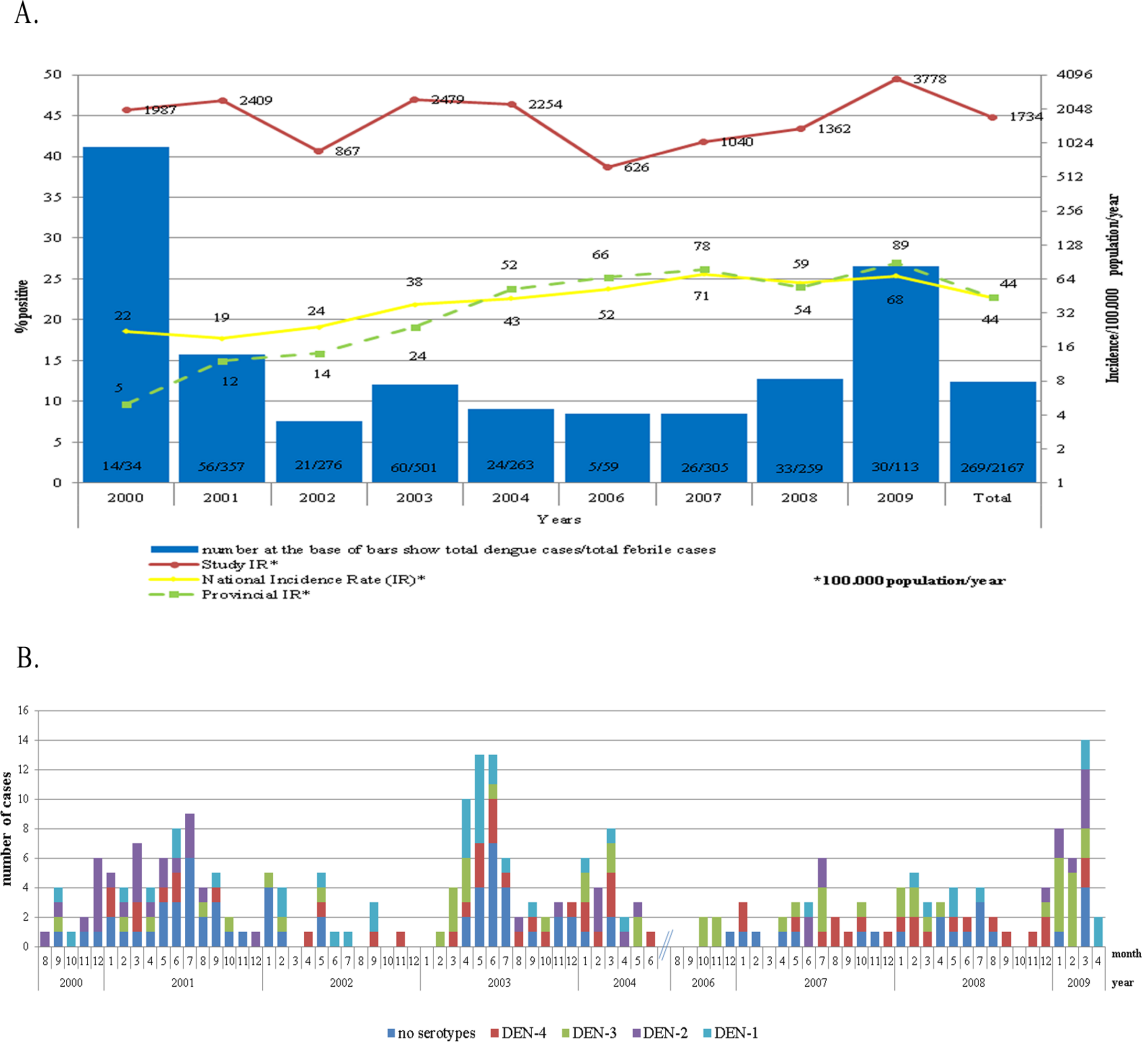
Dengue cases by year, month and serotype distribution, 2000–2004 and 2006–2009. A. Proportion of dengue virus infections among febrile episodes by year (grey bars), incidence rate of dengue virus infections in the cohort (red line), national incidence rate (solid black line) and provincial incidence rate (dashed black line). B. Monthly distribution of dengue cases by serotype.

A total of 2,167 febrile episodes occurred during the course of the study, which encompassed a total of 15,454.5 person months of observation. DENV infections were confirmed in 268 episodes, giving an overall proportion of dengue virus infection among fever patients of 12.4%. This proportion was less than 10% in 2002, 2004, 2006 to 2007, while the highest proportions were observed in 2000 (41.2%) and 2009 (26.6%). The overall incidence rate in the cohort was 1730/100,000 person years. The annual incidence rate was the lowest in 2006 with 630/100,000 person years and the highest in 2009 with 3780/100,000 person years (Fig 2A). The incidence rate was highest in the youngest (18–27 years old) age group (3984/100,000), followed by the 28–37 years old age group (2449/100,000), the >47 years old age group (1661/100,000) and the 38–47 years old age group (1384/100,000). In general, cases started to increase during the rainy season in January, peaked in the first half of the year and then slowly decreased in the second half of the year (Fig 2B).

### Laboratory diagnosis and virological findings

Of 268 symptomatic dengue cases, 92 were confirmed by virus isolation, RT-PCR and serology, 104 by RT-PCR and serology, and 72 only by serological assays. According to HI, IgG ELISA and PRNT antibodies, infections were classified as primary in 21 (7.8%) cases and secondary in 247 (92.5%) cases.In Bandung, all serotypes circulated most years of the study. DENV-2 was absent for 18 months from December 2001 until June 2003. DENV-1 and DENV-2 were not detected for nine months from September 2006 to June 2007 and DENV-2 was absent from August 2007 to November 2008. DENV-3 and DENV-4, conversely, were more evenly distributed throughout the year. From a total of 196 cases where the serotype was identified, DENV-4 was the most frequent (28.6%), followed by DENV-3 (26.5%), DENV-2 (22.4%) and DENV-1 (22.4%).The only month that all serotypes were detected among the cohort simultaneously was in March 2009, at a time during the highest incidence of cases (Fig 2B).

In confirmed cases, IgM antibodies were positive in 7.9% (7/89) of subjects who came to the clinics on day two, 20.2% (18/89) on day three, 36.7% (18/49) on day four and 48.8% (20/41) on day five or more. In 15.74% (42/268) of the cases, IgM antibodies were never detected, not even in the convalescent specimens. All of the cases in which IgM antibodies were never detected were confirmed by HI and in 19 cases were also confirmed by RT-PCR. All of the cases in which IgM antibodies were never detected were secondary infections.

### Sequence analysis

**Fig 3:**
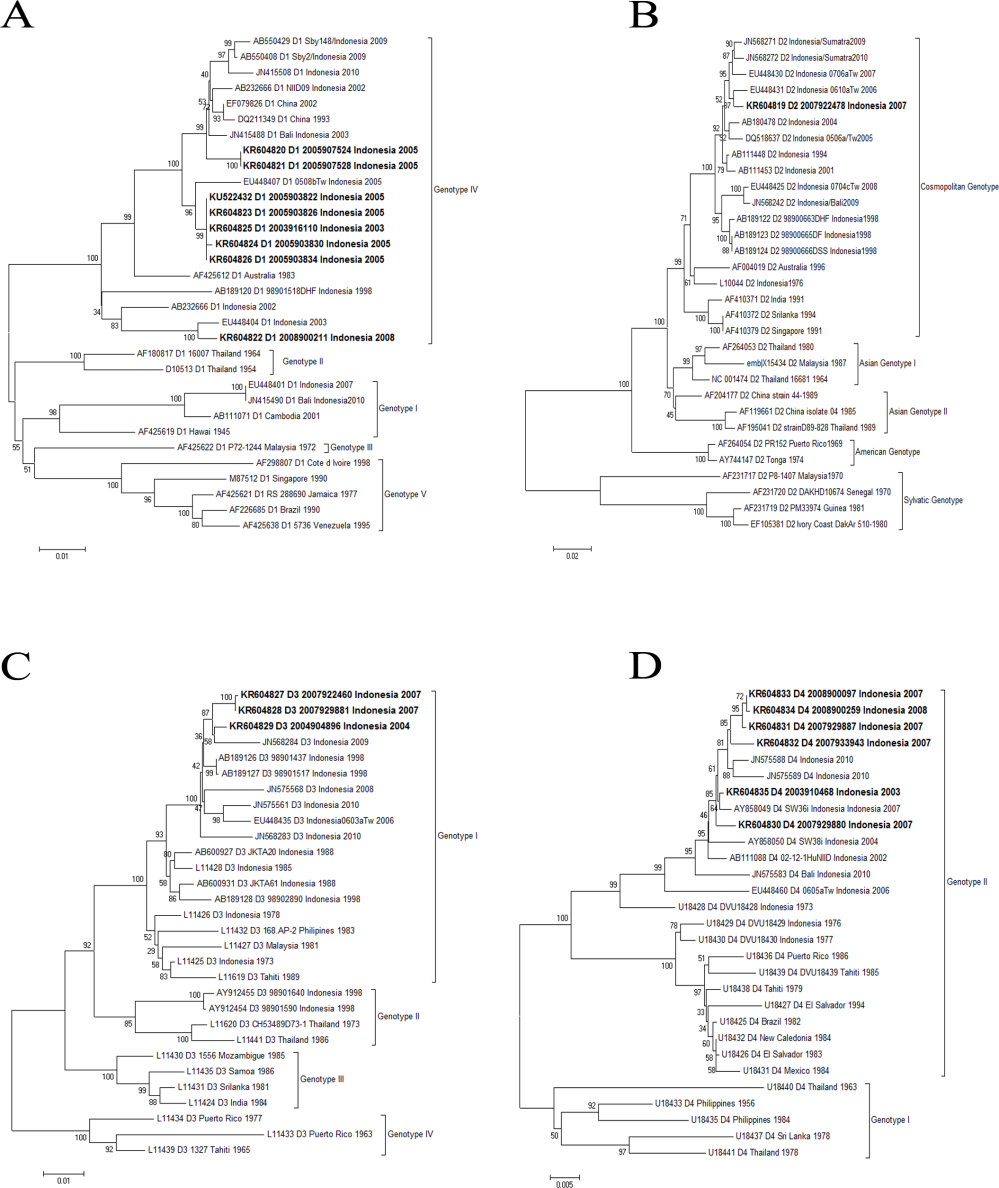
Neighbor-joining phylogenetic trees for the envelope gene from dengue viruses isolated from the cohort. A. Dengue virus-1 B. Dengue virus-2 C. Dengue virus-3 D. Dengue virus-4.

Envelope gene sequences from isolates identified in this study and sequences available in GenBank were used to generate phylogenetic trees for DENV-1, DENV-2, DENV-3, and DENV-4 (Fig 3). The similarity of envelope gene DENV-1 sequences within isolates from this study and other Indonesian isolates were between 98–99% and 94–98%, respectively. The similarity of amino acid sequence within this study and other Indonesian isolates was 98–99% and grouped to genotype IV. The similarity with an Indonesian isolate from 2007 (gb/EU448401) was only 97%, resulting in different genotypes. The similarity of envelope gene DENV-2 sequences with Indonesian isolates from 1976 to 2010 was between 97–98% and the similarity of amino acid sequences was 99%. Genotype analysis grouped this isolate into the Cosmopolitan genotype. Three DENV-3 sequences in the study have similarity of around 96.8–98% with most Indonesian isolates. The similarity in amino acid sequence was 99% and grouped to genotype I. However, the sequence similarity compared to two Indonesian isolates from 1998 (AY912454, AY912455) was only 94.5% and the similarity in amino acid sequences was 98%, resulting in a different genotype. Genotype analysis of five DENV-4 isolates placed them in genotype II, together with other Indonesia isolates from 1973 to 2010.

### Clinical categories, signs and symptoms

The majority of cases were DF (78.4%), followed by DHF grade I (11.9%), DHF grade II (7.5%), unclassified (1.9%) and DSS (0.4%). Since patients were advised to come early when they experienced fever, the mean days from fever onset was 3.2 (±1.2) days, ranging from day two to eight days. Symptoms and signs that were frequently reported included myalgia (91.3%), headache (90.9%), arthralgia (63.8%), nausea (59.6%) and a positive tourniquet test (30.9%). Leukopenia (<4000/mm^3^) was detected in 29% of patients and thrombocytopenia (<150,000/mm^3^) was detected in 34% of patients. Leukopenia and thrombocytopenia by fever day is presented in Table 2. The majority of DF cases presented with an undifferentiated fever; 38.6% (81/210) also presented with hemorrhagic signs or mild thrombocytopenia. There were no cases with complications such as organ impairment.

**Table 2:** Leukopenia and thrombocytopenia by fever day.

Fever day	Leukocytes (<4000/mm3) N = 261	Platelet (<150,000/mm3) N = 258
Day 2	12.4% (11/89)	16/90 (17.8%)
Day 3	21.5% (20/93)	30/92 (32.6%)
Day 4	46.5% (20/43)	16/40 (40%)
Day 5	66.7% (24/36)	25/36 (69.4%)

### Sequential dengue virus infections

Six patients experienced multiple confirmed dengue virus infections resulting in febrile illnesses during their participation in this study. Four of them have been reported elsewhere [33]. For the remaining two, one volunteer had evidence of three sequential dengue infections and one had evidence of two dengue infections (Table 3). The first case was a 33 year old male (ID 50877) with evidence of a previous DENV-4 infection, followed by DENV-1 infection and a DENV-3 infection five and half years later. The clinical diagnosis was DF. The second case was a 28 year old male (ID 50743) with a DENV-2 infection followed by a DENV-3 infection two years later. Clinically, both episodes manifested as DF with hemorrhagic manifestations that required hospitalization. Details for all six sequential dengue virus infections are presented in Table 3.

**Table 3:** Details for sequential dengue virus infections observed in the cohort.

		Study ID					
Episode		50877	50743	50730	51793	52159	52119
1	**Date of illness**	UNK	24/10/2001	UNK	UNK	UNK	UNK
	**Baseline sample date**	3/6/2002	31/7/2001	18/5/2001	30/10/2001	14/8/2001	10/4/2003
	**Acute sample date**	N/A	24/10/2001	N/A	N/A	N/A	N/A
	**Convalescent sample date**	N/A	8/11/2001	N/A	N/A	N/A	N/A
	**Serotype (method)**	UNK	DENV-2(PCR,ISOL)	UNK	UNK	UNK	UNK
	**IgM (acute/convalescent)**	N/A	3.4–8.9	N/A	NA	N/A	N/A
	**HI (acute/convalescent)**	N/A	10–640	N/A	N/A	N/A	N/A
	**PRNT 50%**	DENV-4?	DENV-2	DENV-1?	DENV-2	DENV-2?	?
	**Baseline PRNT 50% (DENV-1,2,3,4)**	<10,<10,10,17	<10,<10,<10,<10	187,<10,12,<10	<10,359,12,11	10,215,<10,<10	<10,124,99,29
	**Acute PRNT 50% (DENV-1,2,3,4)**	N/A	<10,84,<10,<10	N/A	N/A	N/A	N/A
	**Convalescent PRNT 50% (DENV-1,2,3,4)**	N/A	95,2556,15,18	N/A	N/A	N/A	N/A
	**Clinical Category**	UNK	DF+HM	UNK	UNK	UNK	UNK
*2*	***Date of illness***	*21/09/2002*	*02/08/2003*	*6/7/2001*	*5/1/2002*	*21/9/2001*	*6/6/2003*
	***Acute sample date***	*21/09/2002*	*02/08/2003*	*6/7/2001*	*5/1/2002*	*21/9/2001*	*6/6/2003*
	***Convalescent sample date***	*7/10/2002*	*14/8/2003*	*17/7/2001*	*17/1/2002*	*1/10/2001*	*19/6/2003*
	***Serotype (method)***	*DENV-1(PCR*,*ISOL)*	*DENV-3(PRNT)*	*UNK*	*DENV-3 (PCR)*	*DENV-4(PCR*, *ISOL)*	*DENV-4(PCR*, *ISOL)*
	***IgM (acute/convalescent)***	*0*.*9–2*.*8*	*1*.*01–1*.*26*	*0*.*5–4*.*8*	*0*.*7–10*.*9*	*0*.*17–1*	*0*.*2–1*.*1*
	***HI (acute/convalescent)***	*10–5120*	*320–5120*	*<10–640*	*160–10240*	*<10–2560*	*10–2560*
	***Acute PRNT 50% (DENV-1*,*2*,*3*,*4)***	*383*,*234*,*328*,*35*	*243*,*1076*,*349*,*101*	*<10*,*39*,*13*,*<10*	*<10*,*165*,*10*,*<10*	*<10*,*145<10*,*<10*	*<10*,*133*,*<10*,*<10*
	***Convalescent PRNT 50% (DENV-1*,*2*,*3*,*4)***	*14205*,*87886*,*4374*,*162*	*343*,*19493*,*5900*,*154*	*>25000*,*1463*,*6870*,*56*	*3402*,*>1000*, *6042*,*490*	*139*,*3337*, *301*, *493*	*567*,*7404*,*1029*,*5414*
	***Clinical Category***	*DF*	*DF+HM*	*DF*	*DF*	*DF*	*DHF gr II*
3	**Date of illness**	4/4/2008	N/A	7/1/2002	14/7/2003	17/3/2003	25/5/2004
	**Acute sample date**	4/4/2008	N/A	7/1/2002	14/7/2003	17/3/2003	25/5/2004
	**Convalescent sample date**	14/4/2008	N/A	17/1/2002	24/7/2003	27/3/2003	5/6/2004
	**Serotype (method)**	DENV-3(PCR)	N/A	UNK	UNK	DENV-3(RT-PCR)	DENV-3(RT-PCR, ISOL)
	**IgM (acute/convalescent)**	0.5–1.8	N/A	0.48–1.99	1.3–9.3	0.19–1.1	0.4–0.8
	**HI (acute/convalescent)**	80–10240	N/A	40–1280	160–2560	80–5120	80–5120
	**Acute PRNT 50% (DENV-1,2,3,4)**	N/A	N/A	809,76,127,18	77,764,172,137	51,505,57,61	<10,345,<10,<10
	**Convalescent PRNT 50% (DENV-1,2,3,4)**	N/A	N/A	8124,5537,>1000,127	>4500,16750,>1000,2126	>6000,>1000,>6000,>4000	10720,>1000,12103,4146
	**Clinical Category**	DF	N/A	DF	DF	DF	DF

UNK = unknown, ISOL = isolation, DF = dengue fever, HM = hemorrhagic manifestations, DHF = dengue hemorrhagic fever, DHF gr II = DHF grade II

### Clinical severity, serotype, pre-existing immunity and infection sequence

The distribution of clinical severity according to type of infection and infecting serotype is listed in Table 4. Overall, 53 (19.8%) cases were classified as a severe form of illness, DHF I, II and DSS.

**Table 4:** Clinical severity, type of infection and infecting serotype for dengue cases in the cohort.

	Serotypes				
	DENV-1 (44)	DENV-2 (44)	DENV-3 (52)	DENV-4 (56)	Unknown (72)
Primary infections (21)	9	7	4	1	0
DF(+HM)	6(1)	6(1)	1(2)	1	0
DHF I	0	0	0	0	0
DHF II	1	0	1	0	0
Unclassified	1	0	0	0	0
Secondary infections (247)	35	37	48	55	72
DF(+HM)	25(1)	27(2)	26(5)	39(3)	59(5)
DHF I	5	5	10	8	4
DHF II	2	3	7	4	2
DSS	0	0	0	0	1
Unclassified	2	0	0	1	1

DF = dengue fever, HM = hemorrhagic manifestations, DHF = dengue hemorrhagic fever, DSS = dengue shock syndrome

Ninety-six percent of individuals that experienced a severe form of dengue (DHF and DSS) had a secondary infection. The proportion of DHF and DSS among primary cases tended to be lower than secondary cases (9.5% vs 20.6%, p = 0.21). The subjects that experienced a severe illness from a primary infection did not have any co-morbidities. There were 27 cases in which the serotype of the second infecting virus was determined by RT-PCR and the serotype of the previous dengue virus infection could be determined through pre-illness neutralizing antibody titers. Previous infections with DENV-1 were identified in five cases, DENV-2 in 19 cases, DENV-3 in one case and DENV-4 in two cases. Following a DENV-2 infection, infections with DENV-3 or DENV-4 resulted in more severe illness than DENV-1 (40%, 28.6%, and 14.3%, respectively). Two DHF cases occurred when DENV-1 was the first infecting virus (2/5), each followed by DENV-2 or DENV-3, and four cases when DENV-2 was the first infecting virus (4/19), two followed by DENV-3, one DENV-4 and one DENV-1.

## Discussion

This prospective cohort study in West Java provides several important findings on the epidemiology of dengue virus infections in adults living in an endemic area. First, dengue virus is a major etiology of febrile illness (12.4%) in adults in Bandung, West Java, Indonesia. Second, the average incidence rate of symptomatic laboratory confirmed dengue virus infection from 2000–2004 and 2006–2009 was 1734 cases/100,000 person year, or 43 times higher than the district rate (40/100,000 person year) [34]. Lastly, between September 2006 and April 2008 asymptomatic infections were 2.6 times more frequent than symptomatic infections.

The proportion of dengue virus infections among acute febrile patients in the outpatient setting has rarely been reported in Indonesia. A previous study from 1976, which was a bacteriological and serological survey among febrile patients admitted to hospitals in Jakarta, revealed a similar proportion of dengue virus infections [35]. Studies from Malaysia, Myanmar and Thailand report proportions ranging between 5.7 to7%, suggesting dengue is more prevalent in Indonesia [36–38]. The incidence rate in our prospective cohort was calculated from the number of dengue cases that were identified in outpatient clinics, representing both mild and severe cases of dengue, whereas previously reported provincial and national incidence rates from Indonesia were based on the hospitalized cases, representing more severe cases. This could explain why we found an incidence rate 43 times higher than the reported provincial and national figures. This prospective cohort study was conducted in textile factories located in West Java, Indonesia. A limitation of this study is that there is a lack of information specifically about the adult population in Bandung. Thus, while we aimed to represent a working adult population, it is difficult to determine how accurately we did so. While it was not possible to determine where the participants were infected with dengue virus, it seems more likely that the participants were infected outside of the factory as every time a dengue virus infection was confirmed in a cohort member, fogging was immediately conducted at the factory.

Our study revealed that dengue virus infections in adults were mostly uncomplicated DF (78.4%). This finding is different than previous reports that demonstrate severe cases are more predominant [16,17]. One of the reasons for this difference could be that our febrile patients came mostly from outpatients clinics whereas other studies enrolled patients who had indications for hospitalization (usually with platelet count<100,000/mm^3^). Also, we may have under-estimated the number of DHF cases because we relied heavily on serial hematocrit results which may be influenced by intravenous fluid therapy and we used a strict 20% hematocrit increase to confirm hemoconcentration. Although we also performed ultrasonography, at a maximum of once a day, it is probably not sufficient as plasma leakage is transient. It was not possible to classify five cases as the clinical manifestations did not fit with any of the categories. All showed plasma leakage but no thrombocytopenia was noted. For the clinical categories, we also analyzed using the 2009 WHO Criteria that was introduced to answer the difficulties in the use of the 1997 criteria. Our findings revealed that 52 of the 53 DHF cases and the 5 cases that were not possible to be classified would not be considered as severe dengue because the evidence of fluid accumulation was not accompanied by any required respiratory distress [39]. Based on the 2009 criteria, the clinical categories were 148 (55.2%) dengue without warning signs (WWS), 119 (44.4%) dengue with warning signs (WS) and 1 (0.4%) severe dengue. While the 1997 WHO criteria is criticized due to the difficulties in applying it in a clinical setting and the increasing clinically severe dengue cases that did not fulfill the strict criteria of DHF [39], we found that some warning sign criteria in the 2009 WHO criteria are based on clinical judgment and therefore are non-standard (i.e. “severe bleeding as evaluated by clinician”, “abdominal pain or tenderness”, “persistent vomiting”, and “lethargy”). As dengue with warning signs is indicated for hospitalization, researchers have raised concerns about the increasing hospitalization rates [40]. Besides the classical signs and symptoms such as fever, headache and myalgia, we found that leukopenia and thrombocytopenia only supported the dengue diagnosis if tested on day five or more of illness. Furthermore, the development of leukopenia and thrombocytopenia concurred with a sensitivity of DENV IgM antibodies above 50%. We observed an absence of detectable IgM antibodies in dengue cases in some of our participants (15.7%). This phenomenon has been previously reported with the percentages varying from 5.4% [41], 22.1% [42] to 27.6% of convalescent specimens collected on day 7–14 of fever [43].

In our study population, DHF cases were predominantly secondary infections (96.2%). While the proportion of DHF cases due to a secondary infection was higher than in primary infections, the difference was not significant. DHF cases in adults have also been reported in Thailand, Martinique and Pakistan [44–46] In addition, we also identified tertiary infections in six patients, all presenting with DF. This finding, similar to a previous report from Thailand [47], indicates that a previous infection with two dengue serotypes does not necessarily protect an individual against future clinical dengue infections. On the contrary, some level of protection has been demonstrated in several primates studies [48–51], a hospital based study in Bangkok [52], and a prospective cohort in Iquitos, Peru [53].

Our study demonstrated that no serotype was significantly more predominant and most serotypes circulated every year in Bandung. The fact that certain serotypes were not detected for some time could be due to the limited size of the study population. Only during the most intensive transmission period (March 2009) were all serotypes identified in the cohort at the same time. Similar findings have been reported during other outbreaks in Indonesia [16,17].

The sequence of infecting serotypes has been associated with disease severity [54,55]. We found that most cases with well-characterized sequence serotypes had DENV-2 as the previous infecting serotype. Furthermore DHF more frequently occurred when DENV-3 (40%) was the infecting serotype for the second infection compared to DENV-4 or DENV-1, although this difference was not significant. Our data do not support previous findings from Thailand and Cuba that a DENV-2 infection after DENV-1 is a risk factor to develop severe dengue [54,55]. A conclusion cannot be drawn regarding other sequences, as the number of cases was too limited.

We suspect that the majority of asymptomatic infections were the result of DENV-4 infections for the following reasons: first, two of the three primary asymptomatic infections were caused by DENV-4; second, all available pre-illness sera from 24 of 25 secondary asymptomatic infections revealed no or very low neutralizing antibodies to DENV-4; third DENV-4, along with DENV-3, were the predominant serotypes (39.4% and 42.4%, respectively) identified in symptomatic cases during the same period in the same cohort. Asymptomatic or mild forms of disease resulting from DENV-4 infections have been reported before from Indonesia [56] and thought to be the reason for the scarcity of DENV-4 hospitalized cases [32].

Finally, our study shows that there are dynamic changes in the various genotypes of the circulating DENV in Indonesia. DENV-1 genotype IV that was detected in our study in 2003, 2005 and 2008 was first detected in 1968 and since then has been endemic in various areas of Indonesia [57–59]. Regarding other genotypes, DENV-1 genotype I was first reported in Indonesia in 2007 (gb:EU448401) and 2010 in Surabaya [58] and became the predominant genotype in Semarang in 2012, while DENV-1 genotype II has begun to be detected again after it was last detected in 1964 in Thailand [60]. Monitoring the dynamic changes of DENVs is very important as it may relate to clinical severity and thus may have public health impact. For example, despite the high homology of DENV-1 isolates from our study with the strain from the 1998 Sumatera outbreak isolates, no significant rise in cases was reported during our study. A plausible explanation based on the genotype analysis was that the 1998 outbreak was caused by the introduction of DENV-1 genotype IV, but from a different clade. In comparison to the dynamic changes of DENV-1 genotypes, the circulating DENV-2 isolates in Indonesia from 1976 to 2012 and the isolates from our study were only grouped into the Cosmopolitan genotype [57,60]. This Cosmopolitan genotype has previously been associated with severe disease [61]. Similarly, the genotype I of DENV-3 in our study is the common genotype in Indonesia and has remained endemic since 1973. However, during the1998 outbreak, the circulating DENV-3 was from genotype II. Our study was one of only a few studies in Indonesia that has successfully detected and isolated DENV-4 [60]. All DENV-4 isolates were similar to the Indonesia isolates from 1973–2010, belonging to genotype II, which were different to the dominant circulating strain in Thailand (genotype I) [28] and cause mostly mild illness [62]. A limitation of our study is that the viruses selected for sequencing were from a convenience sample and were not selected to be representative. Thus, it is possible that other genotypes of all four dengue viruses may be circulating in this area. Also, the asymptomatic dengue infection was only based on 1.5 years of surveillance. However, this is similar to the ratio from the first two years of the study, using a different approach [25] and another study in Thailand [63].

In conclusion, our study was a population-based cohort study carried out in large factories in West Java, Indonesia with intense monitoring during nearly seven years. We were able to identify dengue virus infections at an early stage and those that presented with minimal symptoms thus providing accurate epidemiological data regarding the spectrum of dengue disease in adults. As dengue is a growing public health threat without effective preventive measures and an unclear pathogenesis of severe illness, further studies examining dengue virus infection in a natural setting need to be conducted.

## Supporting Information

S1 Checklist STROBE checklist. (DOCX)

S1 Form English translation of questionnaire used at the serosurveys. (TIF)

S1 Fig IgG index ratio values by fold increase in HI titer. Sequential serosurvey samples from individuals who did not experience a febrile illness between serosurveys were tested by both HI and IgG ELISA. An IgG index ratio was calculated by dividing the later serorsurvey sample IgG index value by the earlier serosurvey sample IgG index value. All samples with a ≥ 4-fold increase in HI titer were confirmed asymptomatic infections by PRNT.
(TIF)
