# Supplementary figures and images for: The Epidemiology, Virology and Clinical Findings of Dengue Virus Infections in a Cohort of Indonesian Adults in Western Java
Source: PLoS Negl Trop Dis. 2016 Feb 12;10(2):e0004390. doi: 10.1371/journal.pntd.0004390 (PMC4752237; doi:10.1371/journal.pntd.0004390)

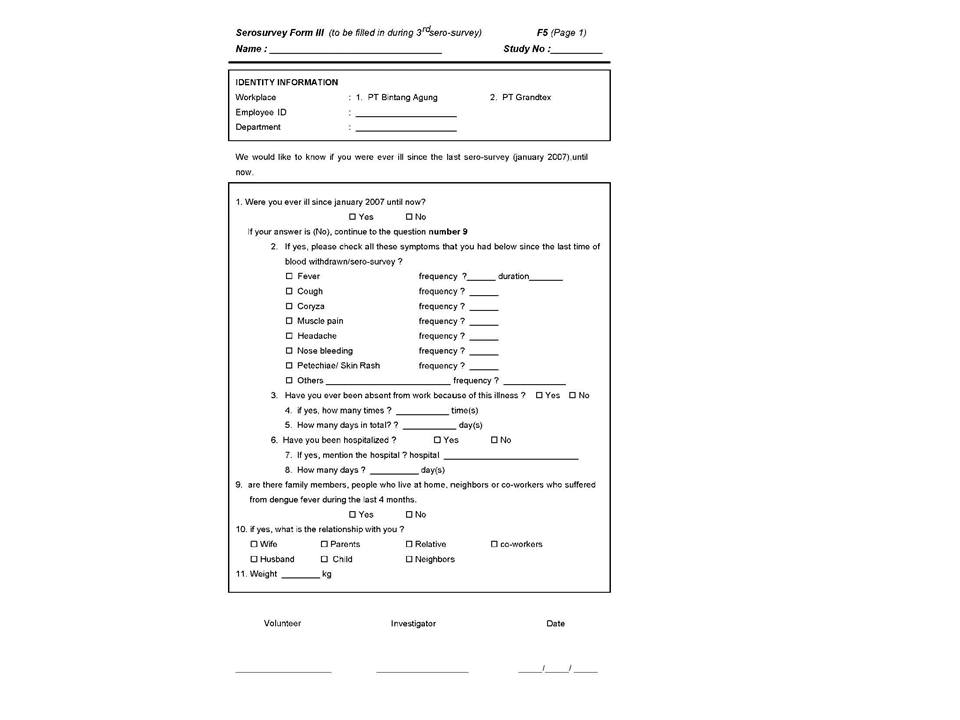

Supplement: S1 Form — (TIF) [file pntd.0004390.s002.tif]

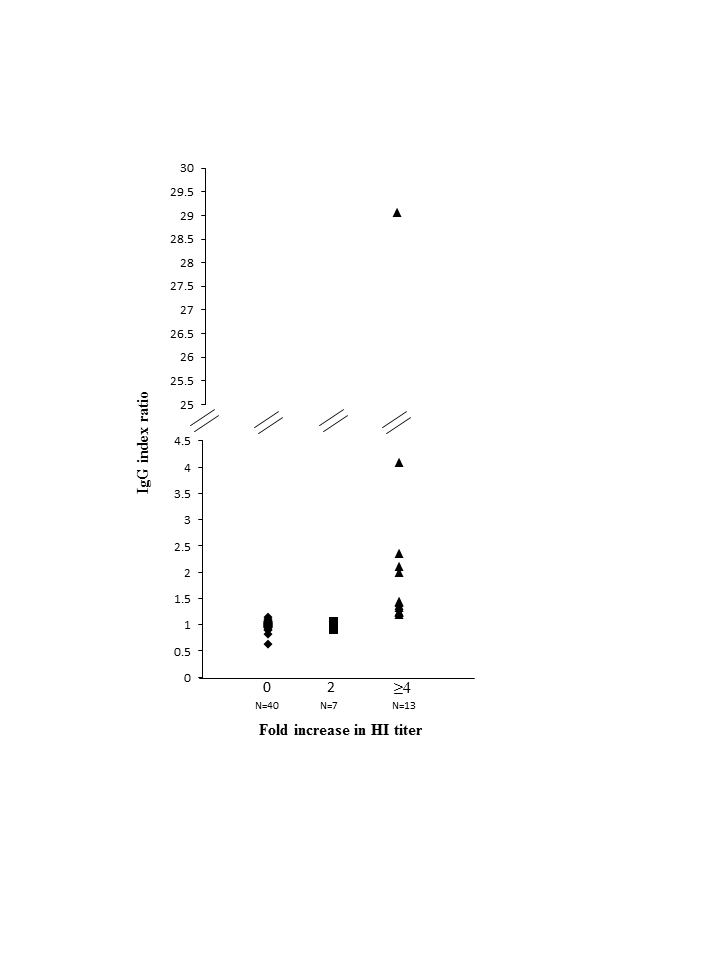

Supplement: S1 Fig — Sequential serosurvey samples from individuals who did not experience a febrile illness between serosurveys were tested by both HI and IgG ELISA. An IgG index ratio was calculated by dividing the later serorsurvey sample IgG index value by the earlier serosurvey sample IgG index value. All samples with a ≥ 4-fold increase in HI titer were confirmed asymptomatic infections by PRNT. (TIF) [file pntd.0004390.s003.tif]
